# An internist's role in perioperative medicine: a survey of surgeons' opinions

**DOI:** 10.1186/1471-2296-9-4

**Published:** 2008-01-21

**Authors:** Lisa PausJenssen, Heather A Ward, Sharon E Card

**Affiliations:** 1 Department of Internal Medicine, University of Saskatchewan, Saskatoon, Canada

## Abstract

**Background:**

Literature exists regarding the perioperative role of internists. Internists rely on this literature assuming it meets the needs of surgeons without actually knowing their perspective. We sought to understand why surgeons ask for preoperative consultations and their view on the internist's role in perioperative medicine.

**Methods:**

Survey of surgeons in Saskatoon, Saskatchewan, Canada regarding an internist's potential role in perioperative care.

**Results:**

Fifty-nine percent responded. The majority request a preoperative consultation for a difficult case (83%) or specific problem (81%). While almost half feel that a preoperative consultation is to "clear" a patient for surgery, 33% disagree with this statement. The majority believe the internist should discuss risk with the patient. Aspects of the preoperative consultation deemed most important are cardiac medication optimization (93%), cardiac risk stratification (83%), addition of β-blockers (76%), and diabetes management (74%).

**Conclusion:**

Surgeons perceive the most important roles for the internist as cardiac risk stratification and medication management. Areas of controversy identified amongst the surgeons included who should inform the patient of their operative risk, and whether the internist should follow the patient daily postoperatively. Unclear expectations have the potential to impact on patient safety and informed consent unless acknowledged and acted on by all. We recommend that internists performing perioperative consults communicate directly with the consulting physician to ensure that all parties are in accordance as to each others duties. We also recommend that the teaching of perioperative consults emphasizes the interdisciplinary communication needed to ensure that patient needs are not neglected when one specialty assumes the other will perform a function.

## Background

There is abundant literature regarding the perceived role of internists in perioperative care. Guidelines for risk stratification, optimization, and prophylaxis are proposed to decrease the morbidity and mortality that can arise from perioperative complications [1-5]. Internists perceive that preoperative consultations are requested to risk stratify and optimize patients for surgery thereby decreasing complication rates. In addition to clinical role models, our own literature teaches us how to be effective consultants [6-11]. Surprisingly, there is little literature or guidance from the surgeons who seek our services as to what they are looking for in an effective consultation.

A multicenter survey of orthopedic surgeons, general surgeons, obstetricians/gynecologists, and general internists revealed significant differences in opinion between surgeons and internists regarding issues such as limiting consultations to a specific question, writing orders on surgical patients, and the concept of comanagement relationships [12].

After discussion with referring services in our own center, we realized there were different perceptions as to the role of internists in the perioperative care of patients. This raised concerns for gaps in care and physician satisfaction as the consultee and consultant are not always aware of each others roles and intentions. Upon reviewing the literature, we discovered little to guide us in this area. Without discussing these issues with each other, the internist frequently proceeds with the consultation and perioperative care based on his or her own assumptions of what is wanted by the surgeon. This may result in suboptimal care of patients.

The objective of this study was to survey the surgeons in our city to understand why they ask for a preoperative consultation and what they envision the internist's role to be in perioperative care.

## Methods

An anonymous survey was sent to all the surgeons in Saskatoon, Saskatchewan, Canada who request general internal medicine consultations from our general internal medicine group. Saskatoon is the referral center for almost 300,000 people in the province and consists of a university-based hospital and two community-based hospitals. More than 30,000 surgeries are performed annually. The protocol was reviewed and approved by the University of Saskatchewan Behavioral Research Ethics Board.

The survey identified the surgeon's specialty followed by five statements outlining an internist's potential role in perioperative care which the surgeons could (strongly) agree or (strongly) disagree with using a five point Likert scale. Surgeons were asked questions focusing on: (1) reason for requesting a consult, (2) the amount of interaction the internist should have with the patient, (3) the role of the internist in the preoperative assessment, (4) the role of the internist in the postoperative care, and (5) when a preoperative consultation should be scheduled. A five-point Likert scale was also used to rank 18 aspects of perioperative care that could potentially involve an internist. The Likert scale indicated 1 (strongly disagree), 2 (disagree), 3 (neither agree nor disagree), 4 (agree), and 5 (strongly agree). Factors were listed to which they responded if they would consult medicine or anesthesia (or both). Lastly, they were asked to write down comments they felt to be important. The survey was developed after discussion within our general internal medicine consultation group of specific patient concerns and noted gaps in care that were repeatedly observed. Exclusion criterion was surgeons in our district who did not routinely request general internal medicine preoperative consults from our group (ophthalmology, and cardiovascular surgery). This is primarily due to distribution of services in the city. Data was tabulated and frequencies calculated on Excel 2003. Median values were calculated using SPSS version 15.0.

## Results

Forty-two of 71 (59%) surgeons responded. Respondents included orthopedic surgeons (9), general surgeons (7), plastic surgeons (4), obstetrician/gynecologists (4), urologists (3), thoracic surgeons (2), vascular surgeons (2), neurosurgeons (2), an otolaryngologist (1), and eight did not specify their area.

The majority of survey questions and their results are outlined in Table 1. The majority of surgeons state they request a preoperative consult for a difficult case (83%) or a specific problem (81%). Sixty-nine percent disagree that it is requested for medico-legal reasons. While half agree that it is to "clear a patient" for surgery, one-third disagree with this statement. In the preoperative period, over 80% believe the internist should discuss the risks with the patient, advise patients of medication changes, and consult other specialties if indicated.

**Table 1:** Responses of Surgeons Regarding Role of Internist in Perioperative Care. (Likert score: 1 – strongly disagree, 2 – disagree, 3 – neither agree nor disagree, 4 – agree, 5 – strongly agree).

	**Median Likert Score**	**(Strongly) Disagree (n = 42)**	**(Strongly) Agree (n = 42)**
**Why do you ask for a preoperative consultation?**			

To "clear" a patient	3	14	19
Specific problem (example diabetes, multiple medications).	4	7	34
Difficult case	5	4	35
Medico legal reasons	2	29	3
Ensure postoperative ward follow-up	3	17	7

**How much interaction should the internist have with the patient?**			

Written impression/recommendation (no discussion with patient)	2	28	12
Inform patient of risk	4	7	33
Advise of medication changes/provide prescriptions.	4	5	35

Postoperatively, only 19% feel that the internist should follow the patient daily while 50% disagree with this. Fifty percent feel the internist should see the patient postoperatively only if called to review. Paradoxically, a majority feel that the internist should adjust medications (eg. insulin, anti-hypertensive pills) postoperatively.

There were discrepant opinions as to whether the internist should inform the patient of risk, with the majority indicating they strongly agreed with this statement but a significant minority (17%) indicating they disagreed or strongly disagreed with the internist informing the patient of risk.

There was no consensus as to when a patient should ideally be seen preoperatively although most agree it should be somewhere between three to seven days and two to four weeks. The following time periods had responses for agree or strongly agree: a) 24% for 24 hours preoperative as the ideal time frame; b) 45% for 3 to 7 days; c) 52% for 2 to 4 weeks and d) 10% for 8 weeks.

**Figure 1:**
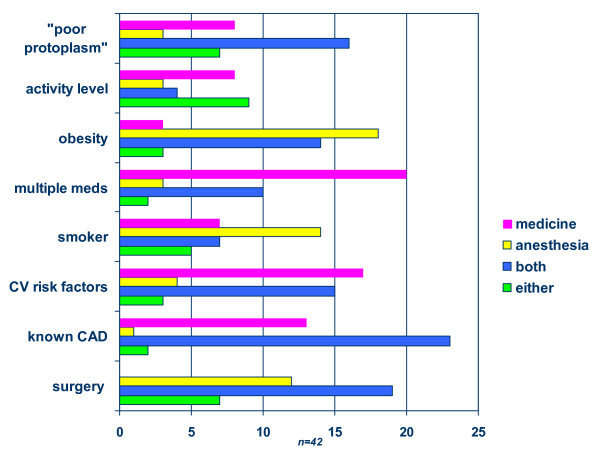
Characteristics that Influenced Preoperative Medicine versus Anesthesia Consultations by Referring Surgeons. (Characteristic versus number of surgeons responding to the question).

The presence of cardiovascular disease, risk factors for cardiovascular disease and multiple medications prompted a medicine consult rather than anesthesia (Figure 1). Obesity, smoking, and type of surgery prompted a consultation to anesthesia. Although obesity and smoking are risk factors for cardiovascular disease, medicine was not preferentially consulted unless there was an established diagnosis.

The aspects of an internist's perioperative care deemed to be most important are: optimization of cardiac meds (93%), cardiac risk stratification (83%), addition of β-blockers (76%), and management of diabetes (74%), respiratory risk stratification (62%), and preoperative medication assessment (62%). Aspects deemed least important are: postoperative family discussions (74%), documentation of allergies (71%), postoperative fluid management (67%), antibiotic prophylaxis (64%), and thromboembolic prophylaxis (60%) (Table 2).

**Table 2:** Surgeons' Perceptions of Aspects of Medicine Consultation Deemed Most or Least Important. ((Likert score: 1 – strongly disagree, 2 – disagree, 3 – neither agree nor disagree, 4 – agree, 5 – strongly agree).

	**Median Likert Score**
**Most Importance**	

Cardiac medication optimization	5
Cardiac risk stratification	5
β-blocker initiation	4
Respiratory risk stratification	4
Preoperative diabetes management	4
Postoperative diabetes management	4
Postoperative cardiorespiratory surveillance	4

**Moderate Importance**	

Postoperative medication surveillance	3
Monitoring drug interactions	3
Family discussions re: risk	3
Management of alcohol withdrawal	3

**Least importance**	

Venous thromboembolic prophylaxis	2
Antibiotic prophylaxis	2
Postoperative fluid assessments	2
Monitoring for alcohol withdrawal	2
Postoperative family discussions	1

## Discussion

In contrast to the abundant literature surrounding internal medicine preoperative assessments, perioperative management of patients, and the effectiveness of a medicine consultation, we could find little literature outlining what surgeons expect from internists regarding perioperative care. Our own anecdotal experience had raised concerns that different specialties had different perspectives as to each others roles and duties leading at times to gaps in care. For example, who would follow through with β-blocker prescriptions or who would inform the patient of specific concerns raised by the internist? These observations prompted a literature review which provided limited guidance to the clinical question of what surgeons expect from internists. This absence of guidance formed the impetus for the present observational study.

Mollema et al retrospectively analyzed the types of requests from surgeons at their site and the impact of the consultation [13]. They stated that 78% of the requests had clear questions although only 29% seemed to ask for a specific recommendation about diagnosis or management while half wanted an "evaluation". In our survey, although half agreed that they ask for a preoperative consult to "clear a patient" for surgery, 81% also felt that they were asking for a specific problem to be addressed. This was a surprising response as anecdotal experience and the literature finds that there is frequently a "general clearance" request as opposed to a specific question stated on the consultation request [8,9]. In Mollema's study performed in an academic center, only 12% of the consultations resulted in a significant change in therapy or outcome. In contrast to the study by Mollema our survey directly asked the surgeons their opinions as to the reason for consultation as opposed to a review of the written consultation. The two studies cannot be directly compared due to the different centers and methodology however in our study the surgeons perceive they are consulting for a specific problem but Mollema found that only 29% of written requests asked for specific recommendations about management and diagnosis of the patient.

Katz et al. surveyed anesthetists and surgeons as to their perspectives as to the purposes and utility of cardiology consultations [14]. This study found substantial disagreement amongst the specialties on the importance and purposes of a cardiology consult. As in our study they found that "clearing the patient for surgery" was not a unanimously accepted reason for the consultation. This study, like ours, found discrepant opinions as to the roles of each specialist. They found that within and between specialties the answers to who is primarily responsible for ensuring that the patient's medical condition is optimized before surgery and who has the primary authority to declare that an elective surgical case may proceed differ. This is similar to our finding that there are discrepant opinions amongst our surgeons as to the degree to which the internist should discuss risk directly with the patient and raises concern that the patient may not be fully informed if each specialty "assumes" it is the other's role.

As surgical techniques have improved over the years and more elderly people are being operated on, the field of perioperative medicine is rapidly growing. The patients' outcomes will depend not only on the science and skill behind the surgery and the preoperative risk assessment, but also must rely on the communication between the surgeon and internist. Without this communication, the internist frequently assumes that his/her opinion is wanted to provide "general clearance" of the patient when in fact the surgeon may have wanted a very specific question to be answered as our results indicate. We agree with the discussion by Katz that such a term is ambiguous and does little to clarify the reason for the consultation for either the internist [14]. As well it does little to ensure that the patient is well served by the consultation. Future perioperative medicine teaching should focus on ensuring that surgeons and internists are able to communicate their mutual meanings behind this term.

There was strong agreement between our surgeons regarding the important aspects of a consultation including cardiac risk stratification, managing medications (including initiating β-blockers), and diabetes management. This is in keeping with the literature aimed at internists regarding preoperative assessments.

As a consultant, we have often been taught that discussions regarding the case should be with the referring physician and only with the patient by prior consent from the referring physician [15,16]. This has been a standard teaching based on the assumption that the surgeon is in a better position to indicate both the benefit of the procedure and combine it with the internist's risk indicated to the surgeon. We often teach our residents that it is not the internist's role to make judgments about whether or not surgery is indicated or even to decide a patient is "too high a risk" for surgery. Most internists do inform patients of their perioperative risk [17] and we found that most surgeons in our city agree with this. Still, a significant minority of surgeons (12/42) felt that the internist should only write down their impression and recommendations and not have any discussion with the patient. Some comments included "...risk of surgery is a discussion with patient/family by the surgeon, not the consultant unless discussed with the surgeon first" and "...discussing surgical risks may confuse/scare patient in whom this has been discussed already." We feel that this is a particularly concerning finding for patients because if both the surgeon and internist assume that the other is informing the patient of particular findings/risks then the patient may never be given essential information. While either perspective of disclosure may be "right" it is critical that the consultant and consultee ensure that their patients are given all information they need to make a decision about their health care choices.

Although half of our surgeons feel that the internist should not routinely follow patients postoperatively, appropriate follow-up may improve compliance with recommendations and detect complications that may otherwise go undetected [14]. As was indicated from the responses in our survey, it is hoped that consultations are requested for difficult or challenging cases or to deal with a specific question rather than for general "clearance" of relatively healthy patients. We also found that surgeons want internists to manage multiple medications and initiate β-blockers if indicated. These scenarios mandate that at least a short period of follow-up ensue to ensure correct medications are administered and to observe for postoperative complications in higher risk patients, especially in the first three to five days. Only eight of our respondents felt that postoperative follow-up by the internist should be routine. In a study of 146 medical consults Katz found that few gave advice that truly impacted perioperative outcome [18]. We postulate that improving the communication between specialties as to each others needs and roles could improve on the benefit to the patient of perioperative consultations.

This study has limitations. First, this is a survey of surgeons' attitudes and may not reflect what they are actually practicing as is evidenced by the fact that they believe they are asking a specific question most of the time (although we perceive this is infrequently written). Surveys with grading systems can be difficult to interpret. Although a space was provided for comments, the surgeons may not have been able to state their exact needs revolving around particular questions. We believe that follow-up discussions will be extremely important to identify surgeons' attitudes and perceptions. Secondly, this survey may not be generalizible to different types of surgical specialties within our community or to other communities. Forty-one percent of the surgeons surveyed did not respond thus limiting the ability to generalize the results. Also, as the sample size in our district is small, we did not differentiate the responses between university-based and community-based or between the various surgical specialties to maintain anonymity. Some groups may have vastly different opinions and needs from others. This has been observed in a study by Salerno et al where it was identified that orthopedic surgeons differed significantly from general surgeons and obstetrician/gynecologists in that they were more likely to prefer a comanagement relationship with internal medicine and wanted internists to broadly manage the patients as opposed to a narrow focus [12]. Although we are not able to indicate what different kinds of surgeons may perceive as the role of internal medicine, we feel that the main message of our study is that each consulting group of general internists should determine their own surgeon's preferences and "assumptions" and not assume from the literature what individual surgeons perceive a consult is for. Lastly, our questionnaire was comprised of questions that we had for our surgeons. It was not intended to be a validated survey that would establish firm roles for either surgeons or internists in our or other centers. Our goal was to have an initial, brief introduction to their opinions so we could begin communicating with each other to improve perioperative care and use the results of this initial survey as a basis for further research exploring the working relationship of general internists and surgeons.

## Conclusion

Out study illustrates the importance of communication and collaboration between specialties to enhance care and ensure the safety of our mutual patients. Without being aware that most surgeons request a preoperative consultation for a specific question or perceived difficult case or whether they want the internist to discuss risk with the patient and actively manage their medications the internist may perform a completely different aspect of care with the patient. These potential gaps in communication could lead to patient safety issues and suboptimal care. We suggest that each internist involved in consultation medicine should discuss with their "consultees' what they desire from the consultation to ensure that they know what is expected of them. For example it should be very clear who will discuss the internist's findings with the patient to ensure that the patient is fully informed. We believe that future research should focus on how to eliminate the gaps in communication and ensure that each specialty has a mutual understanding of each others roles to optimize patient care. In the future teaching of perioperative care indisciplinary collaboration to ensure that the needs of the patient are fully met is vital.

## Competing interests

The author(s) declare that they have no competing interests.

## Authors' contributions

LPJ participated in the conception and design of the study, acquired the data, and drafted the manuscript.

HAW performed the statistical analysis and drafted the manuscript.

SEC participated in the conception and design of the study and drafted the manuscript.

All authors read and approved the final manuscript.

## Pre-publication history

The pre-publication history for this paper can be accessed here:


